# Supplementary material for: Digital hybridity and relics in cultural heritage: using corpus linguistics to inform design in emerging technologies from AI to VR
Source: Int J Digit Humanit. 2026 Apr 1;8(1-2):51–81. doi: 10.1007/s42803-026-00120-4 (PMC13368959; doi:10.1007/s42803-026-00120-4)
Supplement: Supplementary file 1 — (DOCX 29.9 KB) [file 42803_2026_120_MOESM1_ESM.docx]

# APPENDIX A – English Historical Books corpus analysis results

| **#** | **Top 100 modifiers** | **Frequency** | **#** | **Top 100 modifiers continued** | **Frequency** |
| --- | --- | --- | --- | --- | --- |
| 1 | holy | 370 | 51 | like | 7 |
| 2 | sacred | 165 | 52 | goodly | 7 |
| 3 | other | 155 | 53 | many precious | 7 |
| 4 | precious | 131 | 54 | divers | 7 |
| 5 | such | 65 | 55 | poore | 7 |
| 6 | many | 65 | 56 | deare | 7 |
| 7 | Saints | 34 | 57 | various | 6 |
| 8 | small | 33 | 58 | imperfect | 6 |
| 9 | images , | 32 | 59 | suche | 6 |
| 10 | TRUE | 32 | 60 | onely | 6 |
| 11 | FALSE | 30 | 61 | infinite | 6 |
| 12 | popish | 29 | 62 | inestimable | 6 |
| 13 | certaine | 25 | 63 | curious | 5 |
| 14 | superstitious | 24 | 64 | holye | 5 |
| 15 | Romish | 23 | 65 | good | 5 |
| 16 | sad | 22 | 66 | notable | 5 |
| 17 | certain | 22 | 67 | rent | 5 |
| 18 | very | 21 | 68 | Musty | 5 |
| 19 | few | 20 | 69 | special | 5 |
| 20 | dear | 19 | 70 | choice | 5 |
| 21 | old | 19 | 71 | valuable | 5 |
| 22 | great | 19 | 72 | glorious | 5 |
| 23 | rotten | 18 | 73 | more | 5 |
| 24 | only | 17 | 74 | diuers | 5 |
| 25 | miserable | 17 | 75 | paltry | 5 |
| 26 | venerable | 16 | 76 | acid | 5 |
| 27 | scattered | 16 | 77 | little | 5 |
| 28 | famous | 15 | 78 | trifling | 5 |
| 29 | holie | 15 | 79 | invaluable | 4 |
| 30 | poor | 14 | 80 | sole | 4 |
| 31 | ancient | 14 | 81 | outcast | 4 |
| 32 | pretious | 13 | 82 | divine | 4 |
| 33 | new | 12 | 83 | wonderous | 4 |
| 34 | same | 12 | 84 | crosses , | 4 |
| 35 | Martyrs | 12 | 85 | strange | 4 |
| 36 | least | 12 | 86 | wretched | 4 |
| 37 | Religious | 11 | 87 | Church | 4 |
| 38 | dead | 11 | 88 | ashes , | 4 |
| 39 | noble | 10 | 89 | speciall | 4 |
| 40 | several | 10 | 90 | sundry | 4 |
| 41 | last | 9 | 91 | Apostles | 4 |
| 42 | counterfeit | 9 | 92 | louely | 4 |
| 43 | pale | 8 | 93 | rare | 4 |
| 44 | precyous | 8 | 94 | strong | 4 |
| 45 | blessed | 8 | 95 | malignant | 4 |
| 46 | idolatrous | 8 | 96 | worship | 4 |
| 47 | cold | 7 | 97 | lamentable | 4 |
| 48 | principal | 7 | 98 | meer | 4 |
| 49 | mournful | 7 | 99 | choicest | 4 |
| 50 | hallowed | 7 | 100 | Columcille's | 4 |

False hits removed from top 100

| **False hit (item marked as modifier by the software)** | **Frequency** | **Reason for removal** |
| --- | --- | --- |
| d | 24 | not a modifier |
| many other | 13 | already have 'other' |
| theyr | 11 | not a modifier |
| other holy | 9 | already have 'holy' |
| resistentiam | 6 | not a modifier |
| superabit resistentiam | 6 | not a modifier |
| such other | 6 | already have 'other' |
| many holy | 5 | already have 'holy' |
| few trifling | 5 | already have 'trifling' |
| Hauing | 4 | not a modifier |
| dead , | 4 | already have 'dead' |
| Duri , facilè superabit resistentiam | 4 | not a modifier |
| facilè superabit resistentiam | 4 | not a modifier |
| diuers other | 4 | already have 'other' |
| Thys | 4 | not a modifier |
